# The Pathomechanism, Antioxidant Biomarkers, and Treatment of Oxidative Stress-Related Eye Diseases

**DOI:** 10.3390/ijms23031255

**Published:** 2022-01-23

**Authors:** Yi-Jen Hsueh, Yen-Ning Chen, Yu-Ting Tsao, Chao-Min Cheng, Wei-Chi Wu, Hung-Chi Chen

**Affiliations:** 1 Department of Ophthalmology, Chang Gung Memorial Hospital, Linkou Branch, Taoyuan 33305, Taiwan; t6612@seed.net.tw (Y.-J.H.); annie850924@gmail.com (Y.-N.C.); sherry450047@gmail.com (Y.-T.T.); weichi666@gmail.com (W.-C.W.); 2 Center for Tissue Engineering, Chang Gung Memorial Hospital, Linkou Branch, Taoyuan 33305, Taiwan; 3 Department of Medicine, Chang Gung University College of Medicine, Taoyuan 33305, Taiwan; 4 Institute of Biomedical Engineering, National Tsing Hua University, Hsinchu 30012, Taiwan; chaomin@mx.nthu.edu.tw

**Keywords:** oxidative stress, ocular diseases, antioxidant biomarkers, antioxidant therapy

## Abstract

Oxidative stress is an important pathomechanism found in numerous ocular degenerative diseases. To provide a better understanding of the mechanism and treatment of oxidant/antioxidant imbalance-induced ocular diseases, this article summarizes and provides updates on the relevant research. We review the oxidative damage (e.g., lipid peroxidation, DNA lesions, autophagy, and apoptosis) that occurs in different areas of the eye (e.g., cornea, anterior chamber, lens, retina, and optic nerve). We then introduce the antioxidant mechanisms present in the eye, as well as the ocular diseases that occur as a result of antioxidant imbalances (e.g., keratoconus, cataracts, age-related macular degeneration, and glaucoma), the relevant antioxidant biomarkers, and the potential of predictive diagnostics. Finally, we discuss natural antioxidant therapies for oxidative stress-related ocular diseases.

## 1. Introduction

Since the theory of aging being caused by free radicals was published in 1956 [1], various studies have gradually confirmed the dysregulation of oxidative stress as a critical precipitating or exacerbating factor in many pathological processes and the development of diseases. These oxidative stress-related diseases include cancer, cardiovascular diseases, neurodegenerative diseases, diabetes, ocular degenerative diseases, rheumatic immune diseases, and inflammatory diseases [2]. As the main photosensitive organ, the eye directly receives the energy found in sunlight, which travels through the cornea, anterior chamber, lens, and vitreous body to the retina. In addition to causing DNA damage [3], ultraviolet (UV) light can also cause photo-oxidative stress through the production of reactive oxidative species (ROS) [4]. ROS lead to cell damage and aging, resulting in corneal degeneration, lens opacification (cataracts), and the occurrence of eye diseases, including various retinal and optic nerve degenerative diseases, such as glaucoma and age-related macular degeneration (AMD) [5,6,7]. In recent years, with the depletion of the stratospheric ozone layer [8] and the popularity of light-emitting diode (LED) electronic products [9,10], the long-term photo-oxidative stress brought on by the environment to the eyes is bound to increase the prevalence of these degenerative ocular diseases.

## 2. Molecular Aspects of Oxidative Stress-Induced Cell Damage

Oxidative stress in the body comes from the free radicals provided by ROS, reactive nitrogen species, and reactive carbonyl species produced by metabolic or environmental factors; among these free radicals, ROS play the most important role [11]. ROS can compete for the paired electrons of intracellular molecules, leading to lipid peroxidation, protein modification, and chromosomal and mitochondrial DNA (mtDNA) lesions, thereby altering information transmission and gene expression and ultimately causing autophagy, apoptosis, and necrosis and triggering dysfunction in tissues and organs [5,12]. Recent studies have shown that ROS can attack polyunsaturated fatty acids on cell membranes, forming covalent bonds between peroxidized lipids and receptors on the membrane and thus leading to the destruction of the integrity of cell membranes [13]. Peroxidized cell membrane phospholipids can be recognized by scavenger receptors or toll-like receptors and thus induce programmed cell death [14]. Oxidized phospholipids can also activate proinflammatory molecules and thus cause inflammation [15]. The protein modifications induced by ROS can affect the function of structural proteins, the activity of enzymatic proteins, and signal transduction pathways (e.g., redox-sensitive pathways) [16]. ROS can react with nitrogenous bases and sugar-phosphate backbones in DNA, resulting in chromosomal and mtDNA lesions [17]. DNA damage can affect protein-coding regions and noncoding regulatory regions of genes (including untranslated regions and noncoding RNA regions), thereby affecting protein expression and regulation [18,19]. The DNA damage caused by oxidative stress can also induce cell apoptosis through nuclear factor kappa B (NF-κB) pathway activation [20,21]. Additionally, mtDNA damage can affect the respiratory chain, thereby reducing the ability of mitochondria to regulate ROS production. ROS can also affect epigenetic modifications and cause cell aging [22].

**Figure 1:**
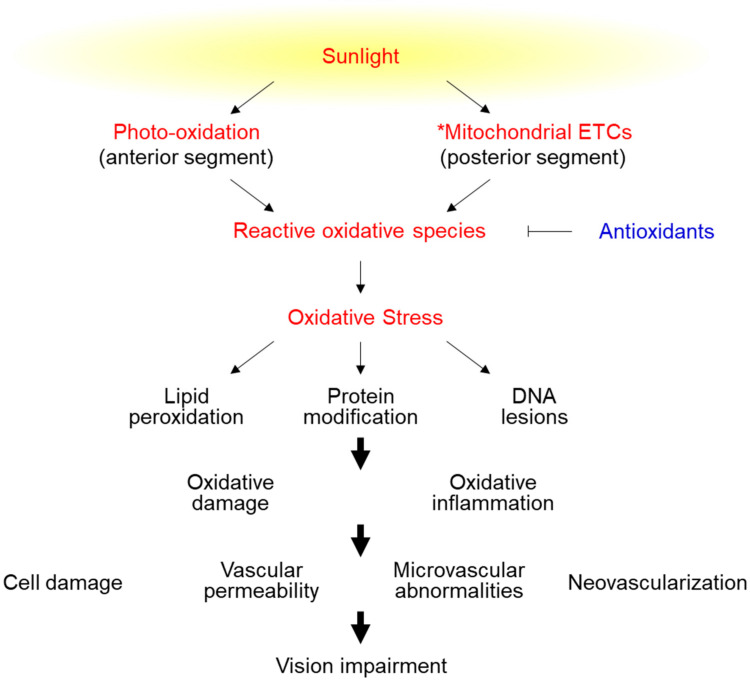
The effects of reactive oxidative species in the eye. The anterior segment includes the cornea, iris, and lens. The posterior segment includes the vitreous humor, retina, choroid, and optic nerve. * Mitochondrial ETCs: Mitochondrial electron transport chains.

Oxidative stress induces autophagy [23]. Lipidated microtubule-associated protein light chain 3 (LC3-II; an autophagosome membrane-bound protein) assists in autophagosome formation, and sequestosome 1 (p62/SQSTM1) binds to LC3-II to assist in autophagosome and lysosome fusion and the formation of autolysosomes to remove abnormal proteins and organelles; this process is called autophagic flux [24]. Severe oxidative stress damage can block autophagic flux, leading to the inability of autophagosomes to bind to lysosomes, resulting in p62 accumulation [25,26]. p62 accumulation induces apoptosis through the activation of caspase-8 [27]. Severe oxidative stress injury can cause cell swelling and mitochondrial swelling, leading to cell membrane rupture and necrosis [28] (graphical abstract and Figure 1).

**Figure 2:**
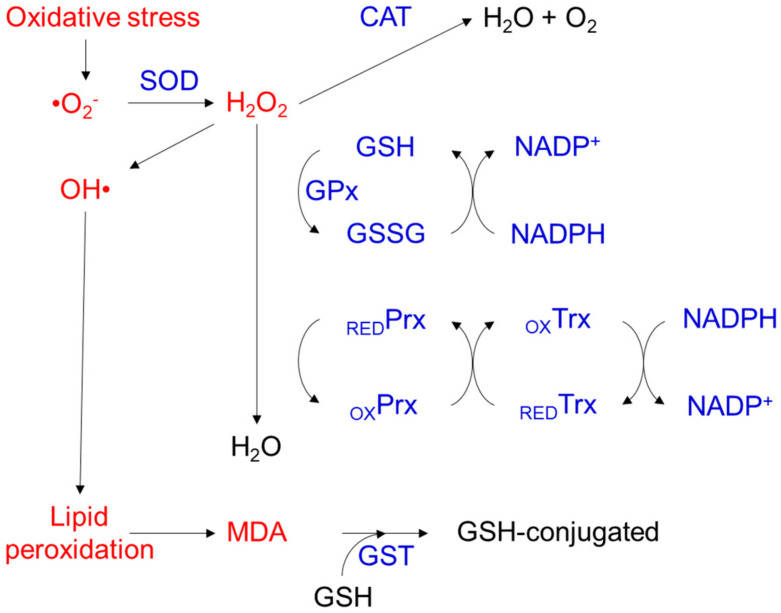
Enzymatic antioxidant defense systems. CAT: catalase; SOD: superoxide dismutase; GPx: glutathione peroxidase; PRX: peroxiredoxin; TRX: thioredoxin; RED: reduced; OX: oxidized; MDA: malondialdehyde; GST: glutathione S-transferase.

To fight against oxidative stress injury, the body has developed antioxidant defense systems composed of antioxidants that inhibit or delay oxidation, thus slowing or eliminating the effects of oxidative stress through prevention, blocking, and repair [11]. The major antioxidant defense system in the human body can be divided into two classes of components: enzymatic and nonenzymatic. Predominantly, antioxidants in the enzymatic system function intracellularly; these enzymes include superoxide dismutase (SOD), catalase (CAT), glutathione peroxidase (GPx), thioredoxin (TRX), peroxiredoxin (PRX), and glutathione S-transferase (GST) [29]. SOD converts superoxide anions (O^2−^) into H_2_O_2_ and oxygen through a disproportionation reaction, and CAT and GPx further catalyze the conversion of H_2_O_2_ into water and O_2_ [30]. The TRX/PRX system acts against oxidative stress through its disulfide reductase activity regulating protein dithiol/disulfide balance [31]. The GST reduces lipid hydroperoxides through its Se-independent glutathione-peroxidase activity [32] (Figure 2). In addition to enzymatic antioxidants, nonenzymatic antioxidants are also vital components. Nonenzymatic antioxidants include both proteins and low-molecular-weight compounds, such as metal-binding proteins (MBPs), ascorbic acid (AA), α-tocopherol (vitamin E), uric acid, and glutathione (GSH). Unlike enzymatic antioxidants, nonenzymatic antioxidants are present not only in cells but also in extracellular fluids, such as plasma, tissue fluid, and cerebrospinal fluid (CSF), where they provide the most significant antioxidant defense mechanism [33,34]. For example, AA is the most important antioxidant in human CSF, and it also plays a pivotal role in defending against the oxidative damage caused by UV light entering the eyes [35]. An increased generation of oxidants (caused by chemical burn, intraocular inflammation or surgery, etc.) or decreased antioxidant capacity (caused by malnutrition or aging, etc.) leads to oxidant/antioxidant imbalances in vivo [36,37,38,39]. Oxidative stress generated by oxidant/antioxidant imbalances results in cell damage and dysfunction.

## 3. Pathogenesis of Oxidative Stress-Related Eye Diseases

**Figure 3:**
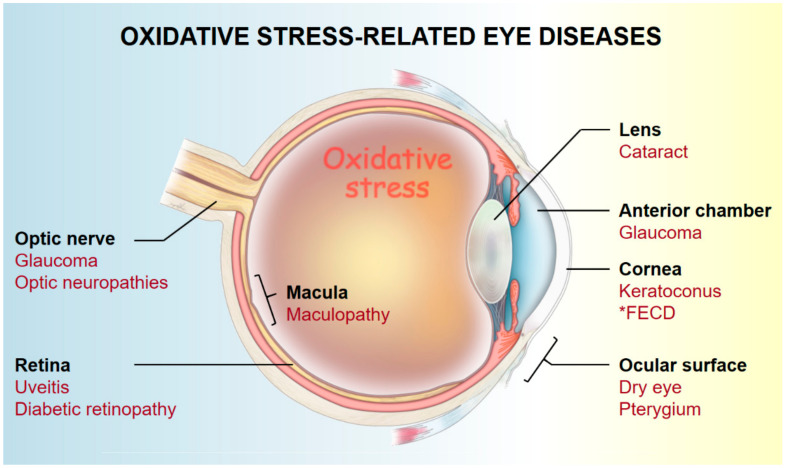
Oxidative stress-related ocular diseases. * FECD—Fuchs endothelial corneal dystrophy.

Oxidative stress is an important pathogenic factor of eye degenerative diseases [5]. Oxidative stress can damage tissues, leading to changes in tissue structure and function, increased vascular permeability, microvascular abnormalities, and neovascularization [16,40,41,42]. In turn, these changes can cause cornea, conjunctiva, and optic nerve lesion formation; lens crystallin denaturation; intraocular pressure (IOP) increase; and retina degeneration [43,44] (Figure 3).

### 3.1. Ocular Surface (Cornea and Conjunctiva)

The cornea and the ocular surface epithelium are exposed to the atmosphere and high concentrations of oxygen (the partial pressure of oxygen is approximately 20%). Therefore, a strong antioxidation mechanism is required to resist oxidative stress. Oxidative damage is associated with the pathogenesis of pterygium, dry eye, keratoconus (KC), and Fuchs endothelial corneal dystrophy (FECD) [5].

Pterygium is a type of cellulite that occurs on the ocular surface. This abnormal tissue proliferation grows from the conjunctiva to the center of the cornea and eventually affects vision. Severe pterygium can interfere with the stability of the tear film and cause dry eye syndrome. 8-hydroxy-20-deoxyguanosine (8-OHdG), an oxidative stress-induced DNA damage marker, is found in pterygium tissue [45]. DNA damage is involved in pterygium inflammation, epidermal hyperplasia, angiogenesis, and lymphangiogenesis [46]. The K-ras oncogene mutation in pterygium may be associated with abnormal tissue proliferation [47]. Malone dialdehyde, a lipid peroxidation marker, is also found in pterygium, resulting in a decrease in the activity of antioxidant enzymes [48]. Oxidative stress can cause Hsp90 overexpression in pterygium epithelium [49]. Hsp90 induces vascular endothelial growth factor (VEGF) expression, and the Hsp90/VEGF pathway is involved in retinal angiogenesis [50], which is probably also related to pathological pterygium angiogenesis [51,52].

Dry eye is caused by the abnormal maintenance of the tear film and is affected by tear secretion and blinking frequency. Tears secreted by the lacrimal glands (LGs) and meibomian glands (MGs) form a film that covers the ocular surface. In addition to lubricating and cleaning, the tear film contains lysozymes and immunoglobulins that can be used for antibacterial and immune responses. The tear film is also rich in antioxidants (e.g., AA, lactoferrin, uric acid, and cysteine). Therefore, tear film dysfunction can lead to infection and inflammation on the ocular surface and cause an increase in oxidative pressure, leading to epidermal and glandular cell oxidative damage, which in turn leads to nonautoimmune dry eye syndrome [53]. In a murine model, aging changed the LG structure and tear secretion, resulting in an increase in oxidative stress markers [54]. The number of 8-OHdG-positive cells in LGs and MGs increases with aging, indicating that oxidative damage is one of the factors that causes gland degeneration seen in aging [55]. ROS have been shown to trigger dry eye through the activation of cytosolic NLRP3 inflammasomes, suggesting that the inflammatory response plays an important role in cell damage caused by ROS [56].

KC is a degenerative corneal disease. The abnormal metabolism of corneal stromal collagen secreted by keratocytes (corneal stromal cells) can lead to a decrease in stromal intensity and affect vision due to the abnormal refraction of the cornea caused by IOP. The oxidative stress-induced DNA damage marker 8-OHdG is increased in KC corneas [57]. ROS can increase the activity of degradative enzymes (matrix metalloproteinases MMP-1 and MMP-2) and reduce the expression of the tissue inhibitors of matrix metalloproteinases (TIMPs) and the secretion of collagen I. These changes reduce the stroma intensity and are related to the occurrence of KC [58,59]. Keratocytes in KC have been shown to exhibit mtDNA damage and mitochondrial dysfunction [60], which is potentially related to their inability to withstand oxidative stress [61]. KC apoptosis induced by oxidative stress is associated with stromal degeneration, thinning, and KC [62,63]. TIMPs are involved in the inhibition of apoptosis. Therefore, the downregulation of TIMPs in KC is also related to keratocyte apoptosis [5]. Autophagy may be another process involved in KC [64]. Oxidative stress induces autophagosome formation [24], and dysregulated autophagy occurs in the corneal epidermis of KC patients [65].

FECD is a degenerative disease that occurs in the corneal endothelial cells (CECs). The corneal endothelium is composed of a monolayer of hexagonal-shaped cells. Human CECs do not have the ability to regenerate. Therefore, when the cells are lost, adjacent healthy CECs can only fill gaps through migration and enlargement, resulting in decreased endothelial cell density (ECD), which in turn leads to a decrease in the water pump function of the corneal endothelium. Several studies have confirmed that the decrease in ECD is related to aging and can also be accelerated by oxidative stress [66,67,68]. Oxidative stress is believed to damage CECs through lipid peroxidation and nitric oxide (NO) production, influencing gene expression and regulating apoptosis through the NF-κB pathway [66,69]. With aging, the ECD in FECD patients declines faster than that in the average person. The pathogenesis of FECD is considered to be multifactorial, and both genetic and environmental factors have been confirmed to be related to the development of this disease [70]. Oxidative stress is an important environmental cause of FECD, and FECD patients have significantly higher corneal endothelial 8-OHdG levels [68]. mtDNA damage caused by oxidative stress leads to a decrease in the number of mitochondria in the corneal endothelium and a decrease in cytochrome oxidase activity, resulting in mitochondrial dysfunction [60]. In FECD patients, compared with healthy individuals, the mtDNA copy number in the corneal endothelium is significantly lower, showing a decrease of 80% [71]. Mitochondrial dysfunction causes CECs to be susceptible to oxidative stress and induces apoptosis. The transcription factor nuclear factor erythroid 2 like 2 (Nrf2), an oxidative stress regulator, is involved in the regulation of lipid peroxidation and is associated with the occurrence of p53/caspase3-dependent apoptosis in CECs [72,73]. The expression of Nrf2 in FECD CECs is significantly lower. Sulforaphane (an agonist of Nrf2) treatment can reduce the loss of FECD CECs by reducing apoptosis [74]. Oxidative stress triggers an autophagic flux blockade in CECs, resulting in p62 accumulation; the accumulation of p62 induces CEC apoptosis through the activation of caspase 8 [26,27]. Peroxiredoxin 1 (PRDX1) is a redox sensor that is involved in the regulation of lipid peroxidation. The significantly lower expression of PRDX1 in FECD patients is associated with nonapoptotic CEC death (ferroptosis) [73].

### 3.2. Anterior Chamber

The anterior chamber contains aqueous humor, which is secreted by the ciliary body and flows through the trabecular meshwork (TM). High IOP caused by aqueous outflow disorders can cause glaucoma. Glaucoma is an optic neuropathy that causes blindness due to the death of retinal ganglion cells (RGCs). An abnormal increase in IOP is the main cause of RGC apoptosis [75]; however, the TM is also susceptible to oxidative damage [76]. Oxidative stress stimulates the activation of the NF-κB pathway in TM cells, inducing apoptosis [77,78]. The oxidative stress-induced mitochondrial injury of TM cells can also lead to changes in apoptosis and TM structure, resulting in increased IOP and disease progression to chronic glaucoma [5,79].

### 3.3. Lens

A cataract is a condition in which the lens becomes cloudy for various reasons and affects vision. Cataracts can be classified as senile or age-related cataracts, juvenile cataracts, or congenital cataracts based on the age of onset. Senile cataracts are the leading cause of vision loss worldwide and the second leading cause of visual impairment [80].

Cataracts form when the crystallin protein inside the lens gradually aggregates after an injury or misfolds to form an insoluble turbid protein that scatters light [81]. Conformational changes in lens proteins are mainly caused by mechanisms, such as oxidative stress, osmotic pressure changes, and phase separation between protein and water [7]. Among these mechanisms, oxidative stress is an important pathogenic mechanism, and the decrease in antioxidant capacity in the lens has been associated with the occurrence of senile cataracts [82]. Either an increase in oxidative pressure in the lens or a decrease in the ability to remove ROS leads to lens opacification [83]. Lipid peroxidation caused by free radicals is the initial mechanism leading to the occurrence of cataracts. Peroxidized lipids can affect the permeability of cell membranes and further change the internal composition and configuration of cells, resulting in the loss of protein function and eventually leading to the occurrence of cataracts [84,85]. Lens epithelial cells maintain the stability and transparency of the environment inside the lens. ROS induce lens epithelial cell apoptosis, producing a large amount of additional ROS, which can cause the degeneration and loss of function of the lens [86]. ROS induce Na,K-ATPase defects on the membrane of lens epithelial cells, a process that can lead to the accumulation of sodium and water in the cells, thereby causing lens opacification [87,88].

### 3.4. Retina and Optic Nerve

To generate and transmit visual evoked potential signals, the retina needs to maintain a high metabolic rate; thus, a large amount of ROS is generated in mitochondria. As such, retinal tissue cells bear higher oxidative pressure than other tissues. Retinal pigment epithelium (RPE) cells maintain the normal function of the retina. In RPE cells, oxidative stress can induce parainflammation [89,90], autophagic cell death [91], and apoptosis [5,92]. Oxidative stress-induced RPE and choriocapillaris damage are associated with AMD [93]. Due to the constant oxidative stress experienced by mitochondria, in the pathological process of AMD, the mtDNA damage caused by oxidative stress is more important than nuclear DNA damage [94]. RGCs are also rich in mitochondria, and mtDNA damage caused by oxidative stress affects the efficiency of the respiratory chain, reduces the production of ATP and the accumulation of ROS, and eventually causes the death of RGCs and the optic nerve [95].

Oxidative pressure increases IOP in the anterior chamber, and the increase in IOP is the main cause of glaucoma, as mentioned in Section 3.2. In addition to harming the TM, the subsequent inflammation and the production of greater amounts of ROS can also cause oxidative damage to the retina and optic nerve and cause glial dysfunction [5]. ROS can trigger autophagy and apoptosis in RGCs and eventually cause optic nerve atrophy and blindness [96,97]. In a glaucoma rat model, through the mitogen-activated protein kinase (MAPK) signaling pathway, ROS generated by the increased IOP induced RGC cell apoptosis through the Bax/caspase-9 pathway [98]. The increase in IOP resulted in the accumulation of LC3II (an autophagosome marker) in the rat RGC layer [99]. Chronic high IOP can cause autophagic flux blockage and p62 accumulation in RGCs, leading to apoptosis [100,101]. Thus, RGC degeneration in glaucoma patients may be related to the unbalanced regulation of autophagic flux, which leads to apoptosis [102].

### 3.5. Others (Oxidative Stress-Related Surgical Complications)

In addition to environmental factors and the dysregulation of antioxidant mechanisms, intraocular surgery itself can also produce oxidative pressure and cause postoperative complications. Intraocular surgery includes cataract surgery [103], beta radiation therapy [104], and laser posterior capsulotomy [105]. It has been reported that intraocular surgery causes a significant decrease in ECD, which is possibly related to the oxidative stress that surgery causes [37,38,39]. Decreased ECD is a significant cause of the loss of function in transplanted corneas, and ECD can predict the success of corneal transplantations [106,107,108]. Patients with a low ECD are contraindicated for the abovementioned surgeries, which can lead to a drastic reduction in corneal cells. Studies have shown that a transplanted cornea with an ECD < 2100 cell/mm^2^ is a risk factor for permanent corneal edema (ECD < 1000 cell/mm^2^) within one year after transplantation [109]. Therefore, in clinical practice, donor corneas with an EDC < 2100 cell/mm^2^ are not considered suitable for transplantation. Even after a successful corneal transplantation, early- or late-stage endothelial cell loss after surgery must be addressed [110] and endothelial cell loss may accelerate [111].

During cataract surgery, phacoemulsification is performed to help remove the degenerated lens. ROS generated by the high-intensity ultrasound oscillations in water during phacoemulsification have been shown to damage the corneal endothelium [37,112]. Due to the high aerobic metabolic activity of human CECs, excess oxidative stress easily damages the DNA and further induces p53 phosphorylation, as well as caspase3-dependent apoptosis [72]. Furthermore, a cell model study found that phacoemulsification triggered apoptosis in bovine CECs. Phacoemulsification-induced caspase3-positive cells could be reduced by the addition of ascorbic acid (antioxidant), indicating oxidative stress involvement in phacoemulsification-triggered apoptosis [113]. The loss of CECs after phacoemulsification can cause corneal edema [114]. Ultrasound emulsification usually causes a CEC loss of 2.5% [115]. However, patients with an insufficient endothelial cell density (iECD) have a more severe cell loss rate of 5.1–12.1%, and they are more prone to pseudophakic bullous keratopathy [116,117].

## 4. Antioxidant Imbalance, Biomarkers, and the Potential of Predictive Diagnostics in Oxidative Stress-Related Ocular Diseases

Enzymatic and nonenzymatic antioxidants comprise the defensive systems against oxidative stress in ocular tissues. The most important enzymatic antioxidants include copper/zinc (Cu/Zn)–SOD (SOD1), manganese (Mn)–SOD (SOD2), CAT, GPx, glutathione reductase, and aldehyde dehydrogenase (ALDH). As nonenzymatic antioxidants, AA (vitamin C), α-tocopherol (vitamin E), GSH, uric acid, tyrosine, and cysteine are regarded as the main ocular antioxidative small molecules [5,118,119]. An imbalance between oxidative stress and antioxidants defense contributes to the pathogenesis of various ocular diseases (Figure 3). Therefore, antioxidants can serve as biomarkers for predictive diagnostics and also as potential therapeutic targets for treating oxidative stress-related diseases [120]. Here, we present representative oxidative stress-related ocular diseases, including KC (cornea), senile cataract (lens), AMD (retina), and glaucoma (anterior chamber and optic nerve), to elaborate the antioxidant mechanisms, biomarkers, and the potential of predictive diagnostics (Table 1).

### 4.1. Keratoconus (KC)

KC is a disease of progressive ectasia of the cornea involving multiple factors [184]. One of the proposed etiologies of KC involves the oxidative stress that the cornea undergoes from constant UV light exposure [118]. Studies have shown dysregulated oxidative stress and antioxidant markers in various samples from patients with KC [185].

#### 4.1.1. Cornea

The total antioxidant capacity (TAC) in the corneas of KC patients is significantly decreased in comparison with the corneas of healthy controls [121]. Corneas from patients with KC exhibited a 2.2-fold increase in *CAT* mRNA levels and a 1.8-fold increase in CAT enzymatic activity in response to the hydrogen peroxide production stimulated by elevated levels of cathepsins V/L2, -B, and -G [122]. The extracellular SOD activity in the central cornea has been found to be halved in KC patients [123]. Expression of NADPH dehydrogenase, ALDH, and heme oxygenase 2 (HO-2) was decreased in the corneal epithelial specimens taken from KC patients [124,125]. The HO-2 enzyme has antioxidative, anti-inflammatory, and anti-apoptotic properties that can defend cells against trauma and apoptotic signals. HO-2 is heavily synthesized in healthy corneal epithelium [126]. In past studies, KC corneas showed an upregulation of ALDH3A1 in the stroma [124] and increased CAT activity in the stromal fibroblasts [63]. One of the proposed pathophysiological mechanisms to explain the decrease in SOD activity in KC is a 7 bp deletion, c.169+50delTAAACAG, located in intron 2 of the *SOD1* gene, which may result in non-functional SOD. However, the use of this mutation as a marker for KC may not be warranted, as the presence of this mutation in KC patients seems rare and varies from study to study [186]. Multiple antioxidant genes regulated by NRF2, including *SOD1*, *GSTM3*, and *HMOX1*, are decreased in patients with KC when compared to those of healthy controls [187]. As for nonenzymatic antioxidants, decreased GSH [121], a reduced glutathione/oxidized glutathione ratio (GSH/GSSG) [188], and stromal serotransferrin [124] have been found in KC corneas when compared with those of healthy corneas.

#### 4.1.2. Tear Film

The TAC of the tear film of KC patients is decreased by 40% compared to that of healthy tear film. Enzymatic antioxidant biomarkers in the tears of KC patients include lower SOD activity and higher GPx activity [127]. It has been suggested that the decreased SOD activity could lead to intense lipid peroxidation, while GPx enzyme activity could increase as a compensatory response to the lipid peroxidation [128]. Among the antioxidants in human tears, AA and uric acid account for up to 50% of the total antioxidant activity [129,130]. Other nonenzymatic small molecule antioxidants in the tear film include GSH, L-cysteine, and L-tyrosine [130]. Among these, increases in tyrosine and uric acid along with decreases in GSH [131] and lactoferrin [132,133] could be used as indicators of KC. Whereas, there was no significant difference in the levels of AA or cysteine between the tear fluids of KC patients and those of age-matched healthy controls [131].

#### 4.1.3. Serum

The TAC of serum from KC patients has not been statistically different from that of normal controls [134,135,136], although significant decreases in serum SOD and GPx, as well as increased CAT activity, have been detected in KC patients [137,138]. The inadequate enzymatic antioxidant defense mechanisms in KC patients can be attributed to *CAT* (rs7943316, A/T) and *GPX-1* (rs1050450, C/T) single nucleotide polymorphisms. Patients with the TT genotype of *CAT* rs7943316 and the T allele of *GPX-1* rs1050450 have lower antioxidant enzyme activities and are at higher risk of developing KC [139,140,141]. The decline in serum GSH levels and the imbalance in the systemic thiol-disulfide homeostasis observed in KC patients suggest the presence of oxidative stress and impaired nonenzymatic antioxidant defense [137,142]. Several trace elements, including Cu, Zn, and selenium (Se), are suggested to have antioxidant activity in the body. The lower serum levels of Zn, Cu, and Se observed in the KC group compared to those of the control group indicate that lower antioxidative activity may be involved in the etiopathogenesis of KC [143,144].

### 4.2. Senile Cataract

Senile cataracts, the progressive opacification of the crystalline lens, result from complicated gene-environment interactions during aging. The cumulative oxidative damage induced by long-term exposure to UV radiation in sunlight, together with the aging antioxidant defense system in ocular tissue, are significant contributory factors in cataract formation [5].

#### 4.2.1. Lens

The crystallins in lens fibers do not regenerate. Accordingly, the human lens is well-equipped with a highly active antioxidant defense system against UV-induced oxidative damage. Interestingly, the oxidative stress in the human lens is mainly managed through scavenging by nonenzymatic antioxidants, especially AA and GSH, rather than by enzymatic antioxidants [5,44,82]. The TAC has been significantly lower in lenses with cataracts as compared to that of the lenses of healthy controls [145]. Moreover, lenses with corticonuclear cataracts have lower TAC than those with subcapsular cataracts [82]. Human cataractous lenses have shown deficiencies in the enzymatic activity of SOD, GPx, and GR, but not of CAT [146]. The reduced enzymatic activity of antioxidants may result from genetic variations in the antioxidant coding genes. To be precise, the G/G genotype of the *SOD1*-251A/G polymorphism may lead to a higher risk of senile cataracts. However, there have been no significant differences in the variant homozygous frequencies of glutathione peroxidase 1 (*GPX1*)-198C/T and *CAT*-21A/T polymorphisms between age-related cataract patients and age-matched healthy controls [147]. In addition, the SOD, CAT, and GPx content in the lenses of senile cataracts have decreased significantly with increasing lenticular nucleus hardness grading [148]. As for the difference in the enzymatic antioxidant level between subtypes of cataracts, patients with cortical cataracts have lower levels of lens SOD than patients with nuclear cataracts [149]. The levels of GSH, AA, and ergothioneine in cataractous lenses are lower than those in post-mortem lenses without cataracts [150]. Whether the concentration of α-tocopherol is higher [151] or lower [145] in cataractous lenses than in control lenses remains controversial. With the progression of senile cataracts, from incipient to mature, the concentrations of AA and GSH are progressively reduced. As for the comparison of nonenzymatic antioxidants among different types of incipient cataracts, the concentration of GSH is higher in lenses with posterior subcapsular cataracts than in lenses with nuclear subcapsular cataracts [82].

#### 4.2.2. Aqueous Humor

The aqueous humor plays a crucial role in protecting the anterior epithelial lining of the lens from oxidative stress. AA is the most abundant and important antioxidant in the aqueous humor, as it is accountable for up to 73.2% of the aqueous humor TAC [119,189]. The TAC in aqueous humor detected in cases with mixed-type cataracts is statistically and significantly lower than the TAC in aqueous humor in cases with cortical and nuclear cataracts [152]. Enzymatic antioxidants in the aqueous humor, including SOD, CAT, and GPx, decrease significantly as lenticular nucleus hardness grading increases [148]. Additionally, patients with cortical cataracts have higher CAT and SOD levels in the aqueous humor than patients with nuclear cataracts [149]. The primary antioxidant component in aqueous humor, namely AA, along with GSH and ergothioneine, is decreased in the aqueous humor of cataract patients compared to that of healthy post-mortem controls [150]. By contrast, a significantly higher uric acid level is detected in the aqueous humor of patients with posterior subcapsular cataracts [153]. In addition, the concentration of AA in the aqueous humor of patients with senile cataracts decreases with age [154]. However, there is no significant difference in AA in the aqueous humor among patients with nuclear, cortical, posterior-subcapsular, or mixed lens opacity [190].

#### 4.2.3. Serum

There is a 30% decrease in serum TAC in cataract patients compared to that of healthy controls [145,155]. Patients with cataracts have lower levels of serum CAT and SOD than healthy controls. Examination of the serum CAT and SOD levels can be an important quantitative indicator for the clinical diagnosis of senile cataracts [149]. Low serum levels of tocopherol [145,155,156], AA [155], GSH [155], and β-carotene [157] are significantly associated with an increased risk for senile cataracts.

### 4.3. Age-Related Macular Degeneration (AMD)

AMD is characterized by progressive degeneration of the macula and is a common cause of blindness in older adult populations. The pathogenesis of AMD is complex, involving metabolic, functional, genetic, and environmental factors; however, as the name implies, degeneration associated with aging is often seen in AMD patients. Major abnormalities of AMD include loss of photoreceptors, degeneration of RPE cells, thickening of the Bruch’s membrane, and thinning of the choroid [158]. Disruption of RPE–extracellular matrix interactions induced by oxidative stress also contributes to the pathogenesis of AMD [159]. Two types of AMD are recognized. Dry-type AMD is characterized by RPE degradation followed by photoreceptor loss in the macular area, which promotes extracellular deposits between the RPE and Bruch’s membrane. This leads to a decreased ability of the RPE to protect the retina. By contrast, wet-type AMD is associated with choroidal neovascularization, which may cause RPE and retinal damage along with exudation, hemorrhages, inflammation, and scar tissue formation in the retina [160].

#### 4.3.1. Retina

Reduced activity of SOD2 and CAT have been found in the RPE and choroid of AMD patients by indirect measurements of Cu and Zn activity [161]. Counterintuitively, a study in 2020 found increased transcriptional levels of the antioxidant enzymes peroxiredoxin 3 (*PRDX3*), *CAT*, and *GPX1* in RPE cells, with decreased complement factor H (CFH) activity, which is commonly seen in the RPE cells of AMD patients [162]. The authors of the study hypothesized that the upregulation of antioxidant enzyme transcription in AMD patients may be due to the inability of the diseased CFH antioxidant pathway to clear oxidative stress. Lutein and zeaxanthin, collectively called the macular pigments, are carotenoids that accumulate in the human fovea. Macular pigments in the RPE absorb high-energy radiation, thereby protecting photoreceptors from oxidative harm. Lower macular pigment optical density (MPOD) in the retina has been shown to be associated with an increased risk of AMD [163]. MPOD is currently being used to assess the efficacy of vitamin supplements for the prevention of macular degeneration.

#### 4.3.2. Serum

A study in 2009 demonstrated lower TAC levels in the serum of patients with exudative AMD compared with those of age- and sex-matched controls [164]. The study reported that the decrease in TAC and increased oxidative stress may result in oxidative damage to lipids, proteins, and DNA. This injurious pathway may lead to some irreversible effects in AMD patients. Multiple studies have shown that the serum samples of AMD patients have decreased SOD, CAT, and GPx activities [165,166]. Antioxidants in serum samples are not a specific pathogenic characteristic of AMD, reiterating the intrinsic decrease in antioxidants as our lives progress. However, the presence of the system-wide deterioration of antioxidants in AMD patients has prompted the hypothesis that variations in antioxidant genes may be identifiable in AMD patients. Macular pigments are less concentrated in the plasma than in the retina, and their association with AMD is also less clear. Of the macular pigments, zeaxanthin appears to be more relevant to the assessment of AMD risk [167]. Decreases in plasma lutein and zeaxanthin seem to increase the risk of AMD [168]. Patients with AMD had significantly lower plasma levels of thiol and native thiol/disulfide ratios (TDRs) compared to those of healthy controls. The plasma native TDR decreased in accordance with the increasing severity of AMD [169].

### 4.4. Glaucoma

The pathophysiology of glaucoma is complex and heterogeneous. There are three main subtypes of glaucoma: open-angle, closed-angle, and normal-tension. Aqueous humor circulation is different in each subtype, which results in varying risk factors and clinical presentations. Despite the complicated etiologies of the different types of glaucoma, disease progression leads to a common pathological state of optic neurodegeneration. Apoptosis of the RGCs is associated with oxidative stress [171]. Levels of oxidative stress are also speculated to have deleterious effects on the TM that drains the aqueous humor from the anterior chamber of the eye [172], which increases IOP and contributes to the development of glaucoma, especially open-angle and closed-angle glaucoma.

#### 4.4.1. Aqueous Humor

A meta-analysis study in 2009 showed the higher oxidative stress markers in the aqueous humor of glaucoma patients, indicating that oxidative stress contributes to glaucoma pathogenesis [173]. However, the change in TAC in the aqueous humor of glaucoma patients is unclear [171]. For example, a study in 2013 showed that TAC in the aqueous humor is decreased in glaucoma patients [170], while another study in 2021 showed an increased outcome [174]. The 2013 study proposed that high oxidative stress was a result of lowered TAC in glaucoma, while the 2021 study hypothesized that high TAC was a reaction to the increased oxidative stress in glaucoma. The varying results and different interpretations further demonstrate the complexity of glaucoma. The increase in oxidative stress can be due to either an increase in IOP or a pathological decrease in antioxidants. Most likely, both interpretations contribute to glaucoma and may be a reason for the contrasting results. Here, we focus on the changes observed for specific antioxidants in the aqueous humor of glaucoma patients. SOD, GPx, and CAT, three common antioxidative markers, were found to be increased in the aqueous humor of glaucoma patients in meta-analyses conducted in 2016 and 2019 [171,173]. It was hypothesized that the increase in antioxidants was a result of the increase in oxidative stress and that antioxidant levels would decrease in the long term. If the assumption is true, it will be unsurprising that other studies have shown SOD1/2 and GST1 to be downregulated in the aqueous humor of open-angled glaucoma patients [176]. One study also found Zn levels to be significantly lower in the aqueous humor of glaucoma patients than those of paired controls [174]. In the same study, magnesium was found to be elevated in open-angled glaucoma patients but not in patients with other subtypes of glaucoma.

#### 4.4.2. Serum

The TAC of the serum of glaucoma patients is decreased when compared to that of healthy controls [170,171,173]. It is hypothesized that damaged TM increases oxidative stress, which in turn recruits antioxidants. As the disease advances, antioxidant capacity dwindles, and a decrease in TAC is observed [175]. It is uncertain whether enzymatic antioxidants, including CAT, SOD, and GPx, increase or decrease in the serum of glaucoma patients, according to the 2016 meta-analysis study [173]. However, the study concluded that the total antioxidant status is lower, which is consistent with the 2013 study. The concentration of serum uric acid is significantly lower in patients with primary angle-closure glaucoma [177,178]. Lower vitamin C levels in the serum have been found in glaucoma patients in multiple studies [179,180,181]. Interestingly, the serum levels of vitamins A, B, and E have not been found to be as strongly associated with the risk of glaucoma as the serum levels of vitamin C, even though a recent meta-analysis showed that the oral supplementation of not only vitamin C, but also vitamins A and E, was beneficial for decreasing glaucoma risk [182]. A study in 2018 found an increase in serum melatonin levels in glaucoma patients [183]. This finding was associated with sleep disturbances and increased oxidative stress caused by glaucoma. The complexity of antioxidants and the pathophysiology of glaucoma have been clearly demonstrated, especially when considering antioxidant levels in the serum. There are no clear-cut increases or decreases in system-wide antioxidants in glaucoma due to multi-system interactions.

### 4.5. The Potential of Predictive Diagnostics for Oxidative Stress-Related Eye Diseases

Endogenous antioxidants play important roles in the prevention of oxidative stress-related diseases and are potential biomarkers for predictive diagnoses. For example, GST has been thought to be one of the most important antioxidants in the lens [191]. In the lenses of patients with senile cataracts, GST levels are significantly lower than those found in healthy controls [150,192]. However, sampling GST from the lens can harm the patient, making antioxidants in lens tissue an unsuitable biomarker for disease detection.

Blood is the most common specimen used for clinical diagnosis. The concentration of serum uric acid is meaningfully different in patients with hypertensive retinopathy [193] and in patients with primary angle-closure glaucoma [177,178]. The concentration of serum zeaxanthin is significantly correlated with MPOD, which highlights the potential of serum zeaxanthin to be used as a biomarker in the management of AMD [194]. Serum TAC, SOD, and GPx are decreased in glaucoma patients [173,195,196]. These serum biomarkers can be measured with enzyme-linked immunosorbent assay (ELISA) and used for diagnosis of the disease.

Being in direct contact with the lens and the cornea, the TAC in the aqueous humor can affect the health of the cornea, anterior chamber, TM, and lens [44,197]. Compared to the blood–retina barrier, the blood–aqueous barrier has better separation properties, causing a greater difference between the blood and aqueous humor than the difference between the blood and the retina circulation. For instance, the concentration of protein in the aqueous humor is approximately hundreds of times lower than that in the plasma, with a predominance of proteins with lower molecular weights [198]. Due to these properties, we propose that aqueous humor samples are better than blood samples for helping with diagnosis. The TAC in the aqueous humor of patients with iECD (<2100 cell/mm^2^) is significantly lower than in that of people with normal ECD [189]. Similarly, lower TAC in the aqueous humor of patients with senile cataracts was observed in our ongoing study (unpublished data). In addition, the TAC in the aqueous humor of patients with glaucoma [170] and diabetic retinopathy [199,200,201,202] has also been found to be significantly lower than that of the healthy population. Ferric reducing antioxidant power (FRAP) assays are often used to assess TAC; however, for the FRAP assay, samples must be diluted to at least 300 μL. This poses difficulties in the analysis of aqueous humor. Although the volume of the human anterior chamber is about 0.3–0.5 mL, the volume of aqueous humor can be safely collected during intraocular surgery is limited (approximately 0.1 mL), which is much less than the volume of blood samples. Therefore, we developed a cupric ion-based TAC (CuTAC) assay for analyzing minuscule sample amounts [189]. With this new assay, samples only need to be diluted to 30 μL instead of the 300 μL used previously, providing a more accurate measurement.

The concentration of AA in the aqueous humor is 20 times higher than that in the plasma [198] (Table 2). AA is thought to be the most critical antioxidant inside the aqueous humor [189]. The AA concentration is lower in the aqueous humor of individuals with senile cataracts than in people without cataracts [150]. Similar to the TAC assay, AA concentration in the aqueous humor can be analyzed with FRAP and CuTAC after the sample is combined with ascorbate oxidases. Other protein antioxidants (e.g., SOD, CAT, GPx) can be used as biomarkers [148,150,153], and these antioxidants can be evaluated using ELISA, similar to what is done for serum samples.

## 5. Natural Antioxidant Therapy for Oxidative Stress-Related Eye Diseases

Natural antioxidant therapy has been widely reported for various ophthalmic diseases, such as dry eye, cataracts, glaucoma, and AMD [205,206,207,208]. The National Eye Institute (NEI) in the United States conducted a large-scale clinical trial (the Age-Related Eye Disease Study; AREDS) in the late 1990s. The trial provided antioxidant vitamins to 3640 AMD patients and 4629 cataract patients. The therapeutic effect of the products was tested. The study found that antioxidant treatment had a significant effect on cataracts and effectively slowed AMD progression [209]. The antioxidant vitamins contained vitamin C, vitamin E, and beta-carotene. Two subsequent reports further investigated antioxidant vitamin treatment for 23,099 AMD patients [210] and 76,756 AMD patients [211], respectively. Although antioxidant vitamins did not prevent AMD, they slowed the progression to advanced AMD and visual acuity loss [210].

Vitamin C (also known as L-ascorbic acid [AA] or ascorbate), due to its water-soluble nature, is more convenient than fat-soluble vitamins for therapeutic applications. Vitamin C is also less prone to side effects caused by excessive accumulation, for example, the risk of beta-carotene causing lung cancer in smokers or the risk of vitamin E causing heart failure in patients with cardiovascular disease or diabetes [210]. Therefore, vitamin C has been studied and used for the treatment of various oxidative stress-related ocular diseases. AA provides antioxidant effects in eye tissues by scavenging free radicals, filtering UV light, and regulating other antioxidant molecules in the eye. As a strong antioxidant, AA can provide electrons to neutralize free radicals in the presence of ROS and is oxidized into ascorbyl radicals. Ascorbyl radicals are relatively stable free radicals, and two ascorbyl radicals react rapidly to become a single AA molecule and one dehydroascorbic acid molecule [212]. On average, the aqueous humor of diurnal animals contains higher levels of AA than that of nocturnal animals [213]. Therefore, AA is considered to play a very important role in the mechanism that protects diurnal animals from eye damage caused by UV light. The UV light-filtering effect of AA in the eye is mainly achieved through absorption, fluorescence quenching, and fluorescence-mediated transformation [214]. AA can significantly reduce UV light in the UVB and UVC bands. Although the ability of AA to absorb UV light in the UVA band is poor, it can significantly reduce the effect of UVA light on the eyes by means of fluorescence quenching and fluorescence-mediated transformation [214]. AA can also assist other antioxidant molecules. For example, AA can assist in the reduction of α-tocopheroxyl radicals to activated α-tocopherol [215], which can inhibit lipid peroxidation.

AA is a water-soluble vitamin, therefore the increase in serum concentration that can be achieved by oral administration is limited, as the excess portion will quickly be excreted through the urinary system. Therefore, for ocular anterior segment diseases (such as corneal defects and iECD), the local administration of AA can effectively increase the local concentration. AA has been used to treat corneal epithelial defects because of its antioxidant properties [216]. In a rabbit model, AA eye drops were shown to help the healing of corneal wounds [217]. AA can prevent lipid peroxidation and CEC apoptosis [66]. It has been reported that the intracameral infusion of AA can improve phacoemulsification-induced CEC loss [218]. We observed that the prophylactical application of topical AA can improve phacoemulsification-induced oxidative stress and loss of human CECs [219]. Furthermore, it also has been shown that oxidative stress-induced cell autophagy and apoptosis can be reversed by AA pretreatment [26].

For the application of oral AA supplements, the antioxidant capacity and the concentration of antioxidants in the plasma or aqueous humor are inverse indicators of the risk of senile cataracts [220]. Supplementation of vitamin C can reduce the risk of cataracts in a population with insufficient antioxidant capacity or low plasma AA concentration [221,222]. However, some studies have indicated that vitamin C supplementation cannot prevent cataracts, slow the progression of cataracts, or reduce the probability of cataract surgery [223,224,225]. These divergent results may be due to the complex causes of cataracts (including UV light, diabetes, metabolic syndrome, drug use, high myopia, and genetics), thus altering the effect of vitamin C antioxidant therapy.

It is difficult to administer drugs specifically to the posterior segment of the eye (currently, only intraocular injection can be used); therefore, oral AA supplementation is still the main method for posterior segment diseases (such as AMD). AMD is closely related to apoptosis caused by oxidative stress. Currently, except for wet AMD with pathological angiogenesis, anti-VEGF treatment can improve hemorrhage and retinal edema, but there is no effective treatment for the progressive apoptosis of retinal cells. Despite the results of large-scale clinical trials (14,236 participants) that found that AA supplementation could not prevent AMD [213], in vitro cell experiments have revealed that pretreatment with AA can help human RPE cells resist oxidative stress [226]. AA can suppress VEGF expression in RPE cells [227]. Therefore, AA treatment may help improve the lesion environment in wet AMD and delay disease progression. Glaucoma can also cause posterior segment damage. At present, the treatment of glaucoma mainly involves drugs, lasers, and surgery to reduce IOP to increase optic nerve blood perfusion indirectly and control related risk factors, such as ROS. However, there is not a consistent conclusion regarding the effect of AA treatment on glaucoma [228]. ROS caused by elevated IOP are an important factor leading to the apoptosis of RGCs. Therefore, by controlling IOP, AA treatment may help delay the degradation of RGCs caused by ROS.

In addition to the antioxidative mechanism of AA, topical AA application significantly increased the ECD of granulomatosis patients with polyangiitis after phacoemulsification in our previous study [229]. It has been further confirmed that noncanonical AA-glucose transporter 1 (GLUT1)–extracellular signal-regulated kinase (ERK) axis-stimulated CEC proliferation may contribute to the corneal endothelium regeneration [230]. AA and valproic acid treatment can activate the proliferative activity of human fetal RPE (fRPE) cells through the regulation of SRY-box transcription factor 2 (SOX2), and activated fRPE cells can repair the damaged retina [231]. Therefore, AA treatment may alleviate oxidative stress-induced ocular tissues damage by promoting cell proliferation and improving tissue regeneration. It has been reported that AA could prolong the replicative lifespan in CEC and adipose tissue-derived human mesenchymal stem cells [232,233]. It has also been shown that AA could maintain the long-term self-renewal in human skeletal mesenchymal stromal cells as well as result in prolonged replicative lifespan and sustained expression of stem cell phenotype in human breast adult epithelial stem cells [234,235]. Adult stem cells have been demonstrated in corneal endothelium, which is derived from ectoderm [236]. Collectively, we speculate that treatment results of AA on corneal endothelial dysfunction could be also related to enhanced adult stem cells, while this viewpoint merits further investigation.

In addition to antioxidant vitamins, many other natural antioxidant compounds (e.g., lutein, zeaxanthin, and curcumin) have been used to treat oxidative stress-related eye diseases [237,238,239]. Lutein, zeaxanthin, and β-carotene are carotenoids present in the retina. The concentration of zeaxanthin is greater than that of lutein in the retinal structure, and zeaxanthin is mainly concentrated in the fovea of the macula. Similar to how the large amount of AA present in the cornea plays an important role in antioxidation, the high concentration of these carotenoids in the retina also indicates that they make significant contributions to antioxidation [240,241]. The addition of lutein and zeaxanthin to the diet can effectively increase MPOD [242]. This may help improve the proinflammatory and proangiogenic profiles of AMD patients [243].

Eye drops containing lutein, zeaxanthin, and curcumin can improve the maintenance of the tear film in dry eye syndrome and relieve inflammation [237,244]. Although curcumin does not exist in the human body, it has been widely used as a treatment and in health care since 1937 because of its various effects (e.g., antioxidative, anti-inflammatory, inhibition of angiogenesis, and promotion of cell proliferation) [245,246,247]. Curcumin has poor solubility and is difficult to add to eye drops. The bioavailability of curcumin has been improved through the development of the curcumin derivative tetrahydrocurcumin (THC) [248]. The solubility, stability, and release rate of curcumin can also be controlled through the use and development of nanocarriers. Curcumin-loaded microparticles have been shown to promote CEC proliferation and resistance to oxidative stress [249]. In a cellular model, the secretion of proinflammatory cytokines from macrophages and abnormal angiogenesis were both inhibited [249]. Curcumin has been shown to inhibit corneal neovascularization via the inhibition of the Wnt/β-catenin pathway [250,251] and to heal the corneal epidermis of diabetic rats [252]. Curcumin can reduce the TM cell damage caused by oxidative stress by inhibiting inflammation-related gene expression, mitochondrial ROS production, and apoptosis and can be used for the treatment of glaucoma [253]. Curcumin protects human retinal epithelial cells against oxidative stress [254,255]. Oral curcumin-based nutritional supplementation can effectively inhibit angiogenesis in neovascular AMD patients [256]. It has been used to treat chronic anterior uveitis and juvenile idiopathic arthritis-associated uveitis [257,258]. The oral administration of curcumin can relieve chronic diabetic macular edema and improve visual acuity [259]. The above reports demonstrate the therapeutic value of natural antioxidants in ophthalmic diseases [248,260].

## 6. Conclusions

Oxidative stress is an important pathomechanism of ocular degenerative diseases. Oxidative stress in the body is regulated by antioxidant mechanisms. Antioxidant imbalances can affect the cornea, lens, retina, and optic nerve and cause such diseases as KC, cataracts, AMD, and glaucoma. Using antioxidant biomarkers, patients with a low antioxidant capacity can be identified, and antioxidant supplementation can be used for disease prevention, delay, or treatment.

## Figures and Tables

**Table 1:** Endogenous antioxidant biomarkers in oxidative stress-related ocular diseases.

Disease	Sample Source	Total Antioxidant Capacity/References (↓): Decreased (--): Not Significantly Different	Antioxidant Biomarkers *		References
			Enzymatic	Nonenzymatic	
Keratoconus	Cornea	(↓) [121]	SOD Catalase Quinone ALDH HO-2	Glutathione	[63,122,123,124,125,126]
	Tear film	(↓) [127]	SOD GPx	Uric acid Glutathione Tyrosine Lactoferrin	[128,129,130,131,132,133]
	Serum	(--) [134,135,136]	SOD Catalase GPx	Glutathione Thiol Copper Zinc Selenium	[137,138,139,140,141,142,143,144]
Senile cataract	Lens	(↓) [145]	SOD GPx GR	Ascorbic acid Glutathione Ergothioneine α-tocopherol	[145,146,147,148,149,150,151]
	Aqueous humor	(↓) [152]	--	Ascorbic acid Uric acid Glutathione Ergothioneine	[150,153,154]
	Serum	(↓) [147,155]	SOD Catalase	Ascorbic acid Glutathione Tocopherol β-carotene	[145,155,156,157]
Age-related macular degeneration	Retina	(--)	SOD Catalase PRDX3 GPx	Lutein Zeaxanthin	[158,159,160]
	Serum	(↓) [161]	SOD Catalase GPx	Zeaxanthin Lutein Thiol	[162,163,164,165,166,167,168,169]
Glaucoma	Aqueous humor	(↓) [170] (--) [171]	SOD GPx Catalase GST	Zinc	[170,171,172,173,174]
	Serum	(↓) [170,171,173,175]	SOD	Uric acid Ascorbic acid Melatonin	[170,173,174,175,176,177,178,179,180,181,182,183]

* Note: This table only includes antioxidants that have been shown to be significantly different in individuals with and without the indicated disease. ALDH, aldehyde dehydrogenase; GPx, glutathione peroxidase; GR, glutathione reductase; GST, glutathione transferase; Quinone, NADPH dehydrogenase; SOD, superoxide dismutase; HO-2, heme oxygenase 2; PRDX3, peroxiredoxin-3.

**Table 2:** The concentration of ascorbic acid (AA) in human ocular tissues.

Tissue	AA Concentration	Compared to Serum	References
Cornea	1.39 ± 0.35 mM	~23 times	[26]
Aqueous humor	1.30 ± 0.62 mM	~22 times	[198]
Lens	2.89 mM	~48 times	[203]
Vitreous humor	1.28 ± 0.37 mM	~21 times	[204]
Serum	0.06 ± 0.03 mM		[203]

## Data Availability

Not applicable.
